# Calcium, phosphate and fluoride ionic release from dental restorative materials for elderly population: an *in vitro* analysis

**DOI:** 10.3389/froh.2025.1609502

**Published:** 2025-08-11

**Authors:** Angelo Aliberti, Franklin Garcia-Godoy, Alexandre Luiz Souto Borges, João Paulo Mendes Tribst, Roberta Gasparro, Mauro Mariniello, Pietro Ausiello

**Affiliations:** ^1^ Department of Neurosciences, Reproductive and Odontostomatological Sciences, University of Naples Federico II, Naples, Italy; ^2^ Interdepartmental Research Centre in Health Management and Innovation in Healthcare (CIRMIS), Naples, Italy; ^3^ Department of Bioscience Research, College of Dentistry-University of Tennessee Health Science Center, Memphis, TN, United States; ^4^ Department of Dental Materials and Prosthodontics, Institute of Science and Technology, São Paulo State University (Unesp), Sao Paulo, Brazil; ^5^ Department of Reconstructive Oral Care, Academic Centre for Dentistry Amsterdam (ACTA), Universiteit van Amsterdam and Vrije Universiteit Amsterdam, Amsterdam, Netherlands

**Keywords:** restorative materials, bioactive dental materials, ionic release, glass ionomer cements, resin composite, fluoride, calcium, phosphate

## Abstract

**Introduction:**

With the increasing prevalence of cervical and root lesions in elderly patients, dental filling materials able to release bioactive ions are gaining importance in the restorative treatment. This study evaluated the release of calcium (Ca^2^^+^), phosphate (PO_4_^3−^), and fluoride (F^−^) ions from different restorative materials (*Equia Forte HT Fil*, *Stela Self Cure* and *Riva Self Cure*) for elderly population through *in vitro* testing conducted under different pH levels and temperature conditions.

**Methods:**

Specimens (10 mm diameter and 2 mm thickness; *n* = 3 for each material) were prepared according to manufacturers’ instructions, immersed in buffer solutions at pH 4.8, 6.8, and 8.8; and stored at 37°C and 44°C. Ion release was measured after 1-, 7-, and 28-days using ion chromatography (fluoride and phosphate ions) and mass spectrometry (calcium ions).

**Results:**

Ion release from dental restorative materials was significantly influenced by pH, temperature, and exposure duration. All materials tested exhibited consistent pH trends, with an increase in acidic conditions and stabilization in neutral and basic environments. Fluoride release was notably affected by pH and exposure time (*p* < 0.001), with *Equia Forte HT Fil* showing the highest fluoride release (34.59 ± 0.63 mg/L) in a basic environment at 37°C. *Stela Self Cure* had a maximum release of 3.55 ± 0.24 mg/L, and *Riva Self Cure* reached 7.67 ± 0.49 mg/L after 28 days at 44°C in an acidic medium. Phosphate release remained stable, while calcium ion release varied significantly among materials. *Stela Self Cure* had the highest calcium release (14.35 ± 0.45 mg/L) in a basic environment, and *Equia Forte HT Fil* showed the highest calcium concentration (30.60 ± 0.73 mg/L) in neutral conditions.

**Conclusions:**

The study highlights the relevance of ion-releasing from some bioactive dental restorative materials commonly employed also for managing Class V cervical lesions and promote remineralization in aging oral environments. Materials tested showed variable fluoride, calcium, and phosphate release depending on pH, temperature, and time. *Equia Forte HT Fil* and *Riva Self Cure* exhibited higher fluoride release, supporting anti-caries potential.

## 1 Introduction

The aging of the population is a global phenomenon that significantly impacts oral health (1). Elderly individuals experience a higher incidence of dental caries and periodontal disease, primarily due to physiological changes such as reduced salivary flow, medications, increased gingival recession, and diminished periodontal support (2, 3). These factors contribute to an elevated risk of both carious and non-carious cervical and root lesions, which pose considerable challenges for restorative dentistry (4).

Periodontal disease, particularly periodontitis, is a chronic inflammatory condition that leads to the progressive destruction of the periodontal ligament and alveolar bone (5–8). It is highly prevalent among older adults, and it is a major risk factor for tooth loss. Gingival recession associated with periodontal disease exposes root surfaces, making them more susceptible to demineralization and to the development of root caries (9, 10). Dental caries is a widespread, biofilm-mediated oral disease resulting from the interaction between acid-producing microorganisms and dietary sugars over time (11). The key culprit is the microbial biofilm that adheres to the enamel surface, with *Streptococcus mutans* playing a central role alongside other species like *Lactobacillus*, *Bifidobacterium*, and *Candida albicans* (12). Root caries is more prevalent in the elderly due to the lower mineral content of dentin compared to enamel, making it more vulnerable to acid attack (13). Furthermore, non-carious cervical lesions (NCCLs), including abrasion, erosion, and abfraction, frequently occur due to mechanical, chemical, and biomechanical stresses, leading to dentin hypersensitivity and structural deterioration (14, 15). Research conducted in the 1970s identified a diverse microbial community through anaerobic culturing of root caries lesions in adults (16). Loesche, Syed, and colleagues identified *Streptococcus mutans*, *Streptococcus sanguinis*, and other streptococcal species, along with *Actinomyces viscosus*, *Lactobacillus*, and *Veillonella* in carious lesions (17–21). The management of root and cervical lesions in elderly patients requires the use of restorative materials that not only provide mechanical stability and durability, but also actively contribute to remineralization and antibacterial protection (22, 23). Conventional restorative approaches include resin composites, glass ionomer cements (GICs), and resin-modified glass ionomer cements (RMGICs), which have been recognized for their fluoride-releasing capabilities (24). The release of bioactive ions such as calcium (Ca^2+^), phosphate (PO₄^3−^), and fluoride (F^−^) is particularly relevant for elderly patients as it enhances remineralization, reduces secondary caries formation, and helps maintain tooth structure over time (25, 26). Fluoride is especially beneficial due to its prolonged release over time, which plays a crucial role in reducing the development of secondary caries (27, 28). Also, calcium ions serve as critical stabilizers within the demineralized collagen matrix, playing a fundamental role in the regulation of mineral homeostasis and structural integrity of dental hard tissues (29). Phosphate ions are integral to the remineralization of dentin, facilitating the binding of calcium ions and promoting the crystallization of apatite (30). The remineralizing and protective effects induced by the ion release from bioactive materials extend beyond the immediate interface of the restoration, influencing distant regions of the oral cavity, including the floor of deep carious lesions exhibiting marginal leakage, the restoration margins, carious and non-carious Class V lesions with exposed cementum (31, 32).

The restorative needs of elderly individuals are further complicated by the presence of a weak acidic oral environment due to reduced salivary flow and buffering capacity (33–35). Moreover, elderly patients often wear removable partial dentures, have poor oral hygiene, and experience increased bacterial biofilm accumulation, necessitating restorative materials with strong antibacterial and remineralization properties (36).

Bioactive restorative materials have gained increasing attention for their potential to address these challenges (37, 38). Glass ionomer cements are widely used due to their hydrophilic nature, fluoride release, and ability to interact with the tooth structure through ion exchange (39, 40). Dental restorative materials, such as *Equia Forte HT Fil*, *Riva Self Cure*, and *Stela Self Cure*, have been developed to combine enhanced mechanical properties with bioactive potential. *Equia Forte HT Fil*, a hybrid GIC, provides superior wear resistance and sustained fluoride release, making it particularly beneficial for high-caries-risk patients. *Riva Self Cure*, a conventional GIC, is known for its strong adhesion to dentin, ease of handling, and prolonged fluoride release, making it suitable for patients with high salivary acidity or poor oral hygiene. *Stela Self Cure*, a self-cure composite, offers improved mechanical strength and wear resistance while incorporating bioactive properties that support long-term remineralization.

Understanding the ionic release properties of these restorative materials under *in vitro* conditions is essential for assessing their potential clinical benefits and longevity. Therefore, the aim of this *in vitro* study was to evaluate and compare the release of fluoride (F^−^), calcium (Ca^2+^), and phosphate (PO_4_^3−^) ions from these three restorative materials under different pH environments (acidic, neutral, basic) and temperatures (37°C and 44°C), to simulate the range of conditions encountered in the aging oral environment. The study also aimed to investigate the impact of these environmental variables on ion release dynamics and to assess the potential of each material to contribute to remineralization and cariostatic activity over time. The null hypothesis was that there would be no significant differences in ionic release between the materials tested, and that changes in pH and temperature would not affect ion release profiles.

## 2 Materials and methods

### 2.1 Sample preparation

Three types of commercially available dental restorative materials commonly used in restorative dentistry were examined. The properties and composition of the materials selected are presented in Table 1. The specimen preparation was carried out according to the manufacturers' instructions. Specifically, *Equia Forte HT Fil* (GC Europe), *Stela Self Cure*, and *Riva Self Cure* (SDI) were processed using self-curing techniques. All materials were shaped using a stainless-steel mold with a diameter of 10 mm and a thickness of 2 mm corresponding to an approximate total surface area of 314 mm^2^ (calculated as: 2πr^2^ + 2πrh). Although individual mass was not recorded, all specimens were prepared using standardized molds and identical volumes of material, following manufacturers’ instructions to ensure consistent geometry and material quantity across groups. Each sample was mixed for 10 s with a 3M ESPE CapMix mixer (3M ESPE, Seefeld, Germany) and immediately placed into the molds. The specimens were gently compressed with a celluloid strip and a smooth condenser to minimize air bubble formation and achieve a uniform surface. No protective coating was applied to the top surface. After 5 min, the specimens were removed and polished with 800-grit abrasive paper using a water-cooled rotating polishing machine (Ecomet 30, Buehler Ltd., Lake Bluff, IL, USA).

**Table 1:** Classification and chemical composition of the tested materials based on the manufacturer's available data.

Material	Manufacturer	Type	Curing mechanism	Composition
Stela Self Cure (LOT n. 1241269)	SDI (Victoria, Australia)	Resin-based restorative material	Self- curing	10–25% UDMA, 5–15% GDMA, 1–10% silica amorphous, 3–7% ytterbium fluoride, 1–5% MDP
Riva Self Cure (LOT n. 1230955)	SDI (Victoria, Australia)	Glass ionomer	Self- curing	20–30%acrylic acid homopolymer, 10–15% tartaric acid, 90–95% fluoride aluminosilicate glass
Equia Forte HT Fil (LOT n. 2309201)	GC Europe (Leuven, Belgium)	Bulk fill glass hybrid	Self-setting	95% fluoride aluminosilicate glass, 5% polyacrylic acid powder, reinforced with silicate particles, 25-< 50% polyacrylic acid, 5-< 10% polybasic carboxylic acid, 5 -< 10% tartaric acid

### 2.2 Material testing conditions and pH measurements

The analyses were conducted using the methodology outlined in previous studies (37, 38). In summary, samples (*n* = 3 for each material) were immersed in 50 ml of buffer solutions at three different pH values (4.8, 6.8, and 8.8) and placed in temperature-controlled laboratory incubators (Precision Thelco, Thermo Fisher Scientific, Waltham, MA, USA) set at 37°C and 44°C. To simulate an acidic environment, a 1 M buffer solution of acetic acid/sodium acetate (CH₃COOH/CH₃COONa·3H₂O) was prepared at pH 4.8. For the neutral condition, a phosphate-citrate buffer at pH 6.8 was employed, prepared using sodium hydrogen phosphate (Na₂HPO₄) and citric acid monohydrate (C₆H₈O₇·H₂O). Additionally, to replicate a basic environment, a 1 M Tris(hydroxymethyl)aminomethane hydrochloride (Tris-HCl) buffer solution was prepared at pH 8.8, utilizing Tris (C₄H₁₁NO₃) and hydrochloric acid (HCl). The materials remained in their respective buffer solutions for 1 day, 7 days, and 28 days before being transferred to 50 ml Falcon tubes for further analysis.

Different specimens were used for each time point. Specifically, three independent samples (*n* = 3) were prepared for each combination of time (1, 7, or 28 days), pH condition, and temperature. This design allowed ion release to be measured from fresh, previously unexposed specimens for each experimental condition. In addition, to prevent ion saturation and maintain a consistent diffusion gradient, the immersion medium was replaced at each time point. Specifically, at each time point (1, 7, and 28 days), the specimens were removed from the solution, and the buffer medium was collected for analysis. The samples were then transferred into fresh buffer solutions corresponding to their assigned pH and temperature conditions for the subsequent immersion period.

For pH measurement and evaluation, 5 ml of the soaking solution was collected from each sample and transferred to 15 ml Falcon tubes. A digital pH meter (Mettler Toledo Seven Excellence pH/Cond Meter S470-Std-K), which had been previously calibrated with standard solutions at pH 4.0, 7.0, and 9.0, was used to conduct the pH measurements.

### 2.3 Assessment of fluoride, phosphate and calcium Ion release

Cumulative fluoride (F^−^) and phosphate (PO₄^3−^) ion release was measured using ion chromatography. For each material sample (*n* = 3 per material), 1 ml of the soaking solution was transferred into a 1.5 ml vial for analysis. The ion concentrations were determined with a DIONEX Integrion HPIC ICS1100 ion chromatography system (Thermofisher, Bremen, Germany), equipped with an IonPac AS27 RFIC (4 × 250 mm) analytical column (Thermofisher). A 25 μl sample was injected at a flow rate of 1.0 ml/min, and ion concentrations were calculated based on the retention times of chromatographic peaks, using calibration curves constructed with standard solutions. Specifically, the calibration curve for F^−^ was prepared with six points at concentrations of 1.0, 2.5, 5.0, 10.0, 25.0, and 50.0 mg/L, while the calibration range for PO₄^3−^ was 0.5–50 mg/L. In detail, the calibration curve included seven points at concentrations of 0.5, 1, 2.5, 5, 10, 25, and 50 mg/L. For F^−^ and PO₄^3−^, the limits of detection (LoDs) were 0.30 and 0.15 mg/L, while the limits of quantification (LoQs) were 1.0 and 0.5 mg/L, respectively.

Furthermore, to assess calcium (Ca^2+^) concentrations, samples of each material (*n* = 3) were immersed in 10 ml of solution and then acidified with a 1% mixture of HNO₃/HCl (3:1 v/v). The samples were analyzed via inductively coupled plasma mass spectrometry (ICP-MS) using a Thermo Scientific ICAP RQ trace elemental analyzer and a Q-ICP-MS system. Data analysis was conducted with Qtegra Intelligent Scientific Data Solution software (version 2.10.3324.131). The instrument's operating conditions were optimized with a tuning solution containing Ba, Bi, Ce, Co, In, Li, and U at 1.00 μg/L (supplied by Thermo Scientific). The measurements were performed in kinetic energy discrimination (KED) mode, utilizing helium as the collision gas. Concentrations were quantified by a calibration curve (CertiPUR, Merck, Darmstadt, Germany) that yielded a correlation coefficient (r^2^) greater than 0.98. Specifically, the calibration range for Ca^2+^ was 1–50 mg/L and it was prepared with six points at concentrations of 1.0, 2.5, 5.0, 10.0, 25.0, and 50.0 mg/L. For Ca^2+^, the limit of detection (LoD) was 0.30 and the limit of quantification (LoQ) was 1.0 mg/L. Moreover, for all analyzed compounds, the relative standard deviation (RSD%) values were below 20%.

Calibration standards for calcium were prepared by serial dilution of the stock solution (1,000 mg/L, Sigma Aldrich, St. Louis, MO, USA), using 1% (v/v) HNO₃ as the diluent. The internal standard solution for ICP-MS analysis was prepared by diluting certified standard solutions of yttrium (Y) at a concentration of 100 µg/ml (Sigma Aldrich, St. Louis, MO, USA). The stock solution was diluted with 1% (v/v) HNO₃ in deionized water to obtain a working internal standard mixture, in which the Y was present at a final concentration of 2,5 mg/L. This internal standard solution was continuously introduced into the ICP-MS instrument via an automated online internal standard addition system, which ensured consistent mixing with blanks, calibration standards, and samples at a fixed 4:1 ratio using a peristaltic pump. In contrast, for the analysis of anionic species such as phosphate (PO₄^3−^) and fluoride (F^−^), ion chromatography (IC) was employed. Calibration standards for these anions were prepared by appropriate dilution of certified stock solutions (1,000 mg/L, Sigma Aldrich, St. Louis, MO, USA) with bidistilled water.

**Figure 1:**
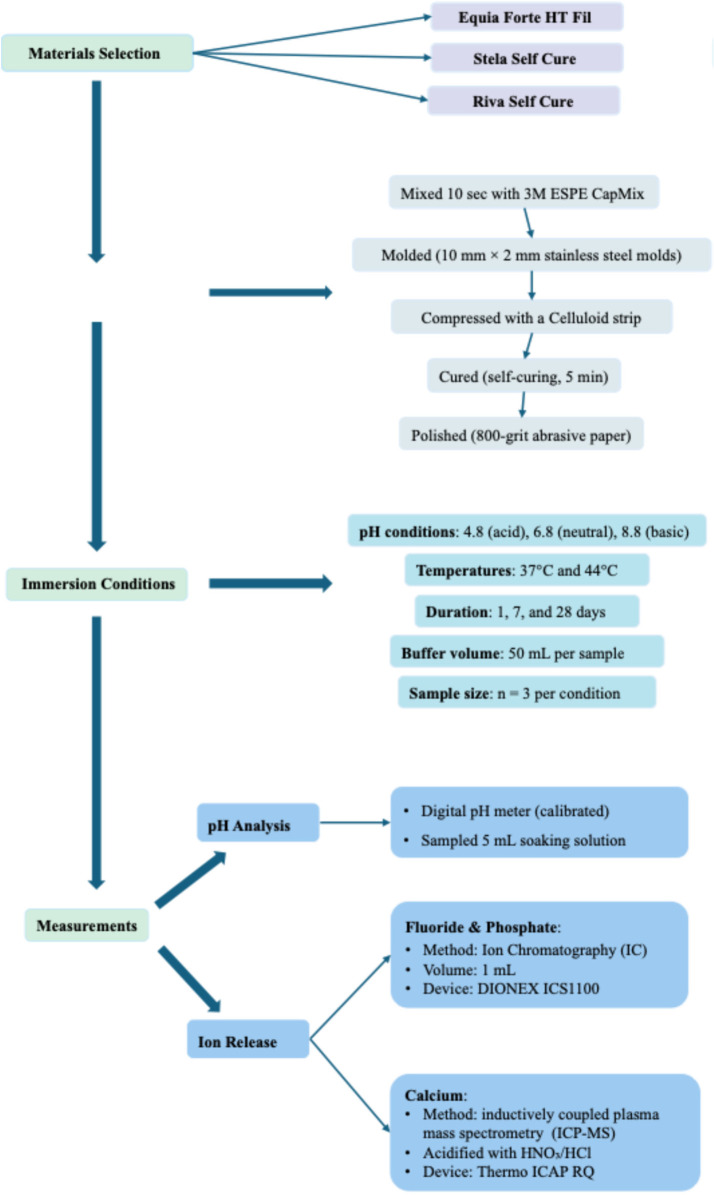
Study design flowchart illustrating the experimental allocation. *Equia Forte HT Fil*, *Stela Self Cure* and *Riva Self Cure* were tested under three pH conditions (4.8, 6.8, 8.8), at two temperatures (37°C and 44°C), and at three time points (1, 7, and 28 days). For each combination of time, pH, and temperature, three independent specimens (*n* = 3) were used. Ion release of fluoride (F^−^), calcium (Ca²^+^), and phosphate (PO₄³^−^) was measured accordingly.

The sequence of experimental procedures, including sample allocation across different pH values, temperatures, and time points, is summarized in the flowchart presented in Figure 1.

### 2.4 Statistical analysis

*a priori* power analysis was performed using G*Power (version 3.1.9.7) to determine the adequacy of the sample size for detecting differences in ion release across materials and timepoints. A repeated measures ANOVA with three groups (materials) and three timepoints (1,7 and 28 days) was considered, with an assumed medium-to-large effect size (f = 0.4), a significance level (*α*) of 0.05, and a power of 80%. The analysis indicated that a minimum total sample size of 27 was sufficient to detect statistically significant effects. Therefore, three replicates per material per condition were included, meeting the required sample size for the expected effect size.

Statistical analyses were conducted using STATA 14.0 software (College Station, TX, USA). Descriptive statistics were applied to calculate the mean concentrations, standard error, and the minimum and maximum values for each material. The Shapiro–Wilk test was used to assess the normality of variable distribution. To evaluate intergroup differences based on pH, temperature, and exposure time, mixed-effects models (MEMs) were employed. The statistical significance of each independent variable was determined using the Wald test, with a significance level set at *p* < 0.05. In the mixed-model ANOVA applied in this study, the variables pH, temperature, pH after immersion, and immersion time were treated as fixed effects, as they represent controlled experimental conditions whose influence on the dependent variables was directly assessed. In contrast, the variable type of material was specified as a random effect. This choice was made to account for the variability associated with the different restorative materials included in the study, which are considered as a random sample drawn from a broader population of similar materials. By modeling type as a random effect, the analysis appropriately captures between-group variability and allows for generalization beyond the specific materials tested. This mixed-effects structure provided a more accurate and reliable estimation of the fixed effects while controlling for random variation across material types.

To account for repeated measures and within-group correlation, data were analyzed using a linear mixed-effects model with material type included as a random effect. This statistical approach inherently manages multiple comparisons, eliminating the need for additional *post hoc* tests.

## 3 Results

### 3.1 Results of pH measurements

**Figure 2:**
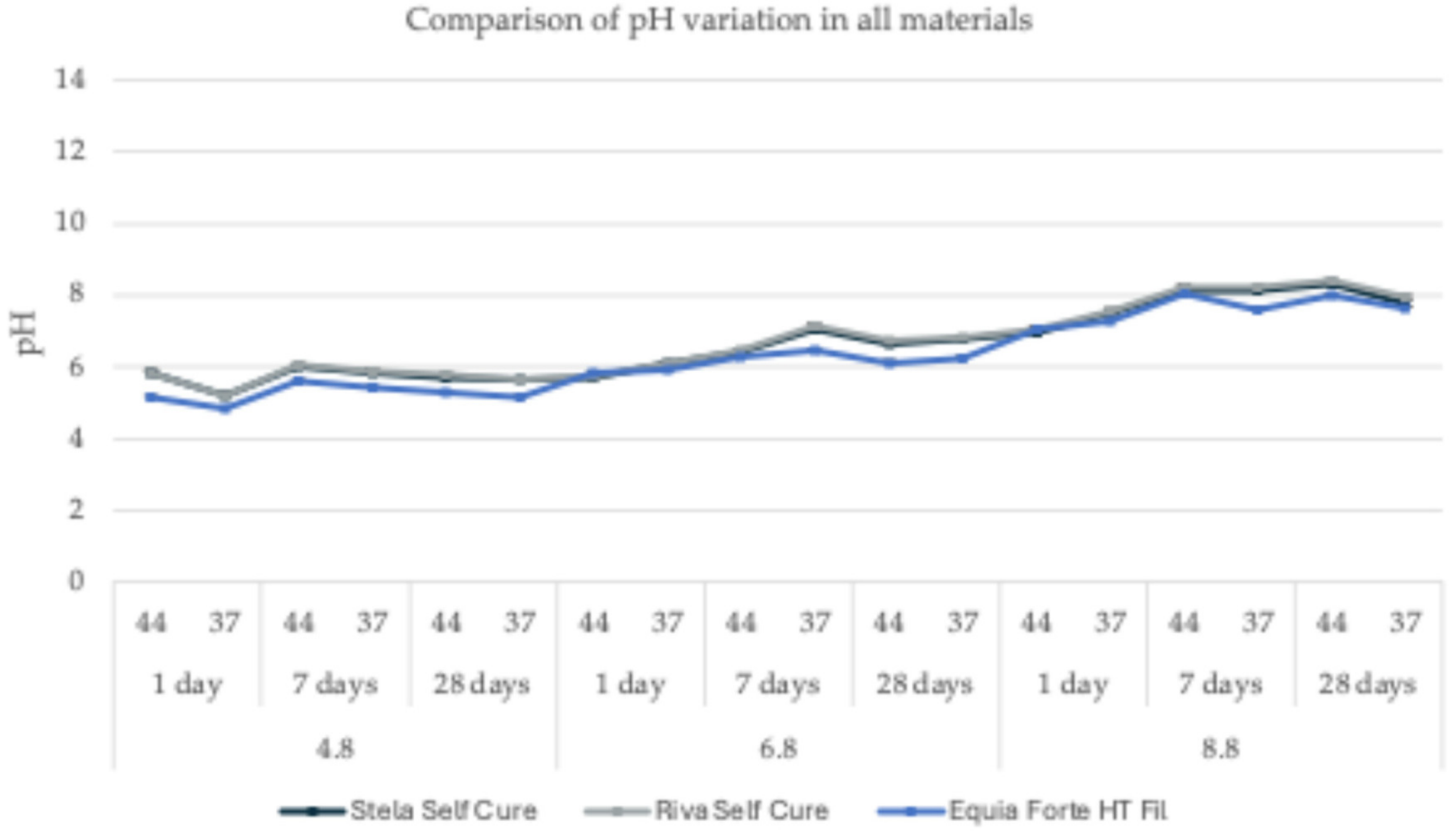
pH variation comparison for the three tested materials in buffered solutions with pH values of 4.8, 6.8, and 8.8, measured at two temperatures (37°C and 44°C) across three observation periods (1, 7, and 28 days).

Figure 2 illustrates the pH variations observed across different materials under controlled experimental conditions, which included acidic, neutral, and basic environments, two temperature settings (37°C and 44°C), and three observation periods (1, 7, and 28 days). A detailed summary of the results is provided in Table 2.

**Table 2:** The mean pH values and corresponding standard deviations (SD) of the soaking solution following the immersion of each tested material (*Equia Forte HT Fil, Stela Self Cure, Riva Self Cure*) in different environments (acidic, neutral, and basic) were measured at two temperatures (37°C and 44°C) over three observation periods (1, 7, and 28 days).

Buffer solution pH	Time	T (°C)	pH values		
			Stela Self Cure	Riva Self Cure	Equia Forte Ht Fil
4.8	1 day	44	5.83 ± 0.05	5.85 ± 0.08	5.15 ± 0.07
		37	5.22 ± 0.03	5.22 ± 0.07	4.87 ± 0.03
	7 days	44	6.01 ± 0.07	6.05 ± 0.08	5.63 ± 0.04
		37	5.85 ± 0.05	5.90 ± 0.06	5.42 ± 0.07
	28 days	44	5.69 ± 0.03	5.78 ± 0.05	5.28 ± 0.03
		37	5.65 ± 0.05	5.65 ± 0.03	5.18 ± 0.05
6.8	1 day	44	5.71 ± 0.06	5.75 ± 0.07	5.84 ± 0.04
		37	6.10 ± 0.07	6.12 ± 0.04	5.92 ± 0.03
	7 days	44	6.41 ± 0.04	6.45 ± 0.05	6.29 ± 0.04
		37	7.05 ± 0.04	7.15 ± 0.08	6.48 ± 0.04
	28 days	44	6.63 ± 0.03	6.73 ± 0.05	6.13 ± 0.07
		37	6.77 ± 0.07	6.85 ± 0.03	6.26 ± 0.08
8.8	1 day	44	6.95 ± 0.06	7.08 ± 0.06	7.08 ± 0.05
		37	7.50 ± 0.03	7.55 ± 0.08	7.28 ± 0.08
	7 days	44	8.16 ± 0.05	8.25 ± 0.07	8.05 ± 0.07
		37	8.12 ± 0.05	8.22 ± 0.04	7.59 ± 0.08
	28 days	44	8.33 ± 0.04	8.40 ± 0.05	8.01 ± 0.03
		37	7.78 ± 0.07	7.94 ± 0.06	7.64 ± 0.05

All tested materials exhibited similar trends, with only slight variations in pH values. Under acidic conditions (pH = 4.8), a general increase of approximately one pH unit from the initial values was observed (Figure 2). Specifically, for *Equia Forte HT Fil*, pH values ranged from 4.87 ± 0.03 at 37°C after 1 day to 5.63 ± 0.04 at 44°C after 7 days, with an average of 5.25 ± 0.26. Likewise, *Stela Self Cure* showed pH values between 5.22 ± 0.03 at 37°C after 1 day and 6.01 ± 0.07 at 44°C after 7 days, with a mean of 5.71 ± 0.27. Similarly, for *Riva Self Cure*, pH values ranged from 5.22 ± 0.07 at 37°C after 1 day to 6.05 ± 0.08 at 44°C after 7 days, resulting in an average of 5.74 ± 0.29.

In a neutral environment (pH = 6.8), the variations in pH were minimal (Figure 2). However, unlike in acidic conditions, an initial decrease of approximately one pH unit was observed within the first 24 h, followed by stabilization. Specifically, for *Equia Forte HT Fil*, pH values ranged from 5.84 ± 0.04 at 44°C after 1 day to 6.48 ± 0.04 at 37°C after 7 days, with an average of 6.15 ± 0.24. Similarly, *Stela Self Cure* exhibited pH values between 5.71 ± 0.06 at 44°C after 1 day and 7.05 ± 0.04 at 37°C after 7 days, resulting in a mean value of 6.45 ± 0.48. For *Riva Self Cure*, pH values varied from 5.75 ± 0.07 at 44°C after 1 day to 7.15 ± 0.08 at 37°C after 7 days, yielding an average of 6.51 ± 0.51.

Under basic conditions (pH = 8.8), the trend was like that observed in the acidic environment, with an initial decrease of approximately two pH units within the first 24 h, followed by a gradual return to the initial value (Figure 2). For *Equia Forte HT Fil*, pH values ranged from 7.08 ± 0.05 at 44°C after 1 day to 8.05 ± 0.07 at 44°C after 7 days, with an average of 7.61 ± 0.39. In the case of *Stela Self Cure*, pH values varied between 6.95 ± 0.06 at 44°C after 1 day and 8.33 ± 0.04 at 44°C after 28 days, yielding a mean of 7.81 ± 0.52. Similarly, for *Riva Self Cure*, pH values ranged from 7.08 ± 0.06 at 44°C after 1 day to 8.40 ± 0.05 at 44°C after 28 days, with an average of 7.91 ± 0.50.

Overall, while minor differences were observed among the tested materials, the general trend remained consistent. Under acidic conditions, all materials exhibited an increase of approximately one pH unit, whereas in neutral and basic environments, an initial decline in pH was followed by stabilization or a return to baseline. These findings indicate that the materials maintain relatively stable pH profiles over time under different environmental conditions, with slight variations reflecting their individual responsiveness to pH changes. Such insights are valuable for evaluating their long-term performance and potential suitability for restorative dentistry, particularly in elderly patients.

### 3.2 Fluoride and phosphate Ion release

The release of fluoride and phosphate ions from all tested materials under three different pH conditions (4.6, 6.8, and 8.8), two temperatures (37°C and 44°C), and three observation periods (1, 7, and 28 days) is presented in Figuress 3,4 and detailed in Tables 3,4.

**Figure 3:**
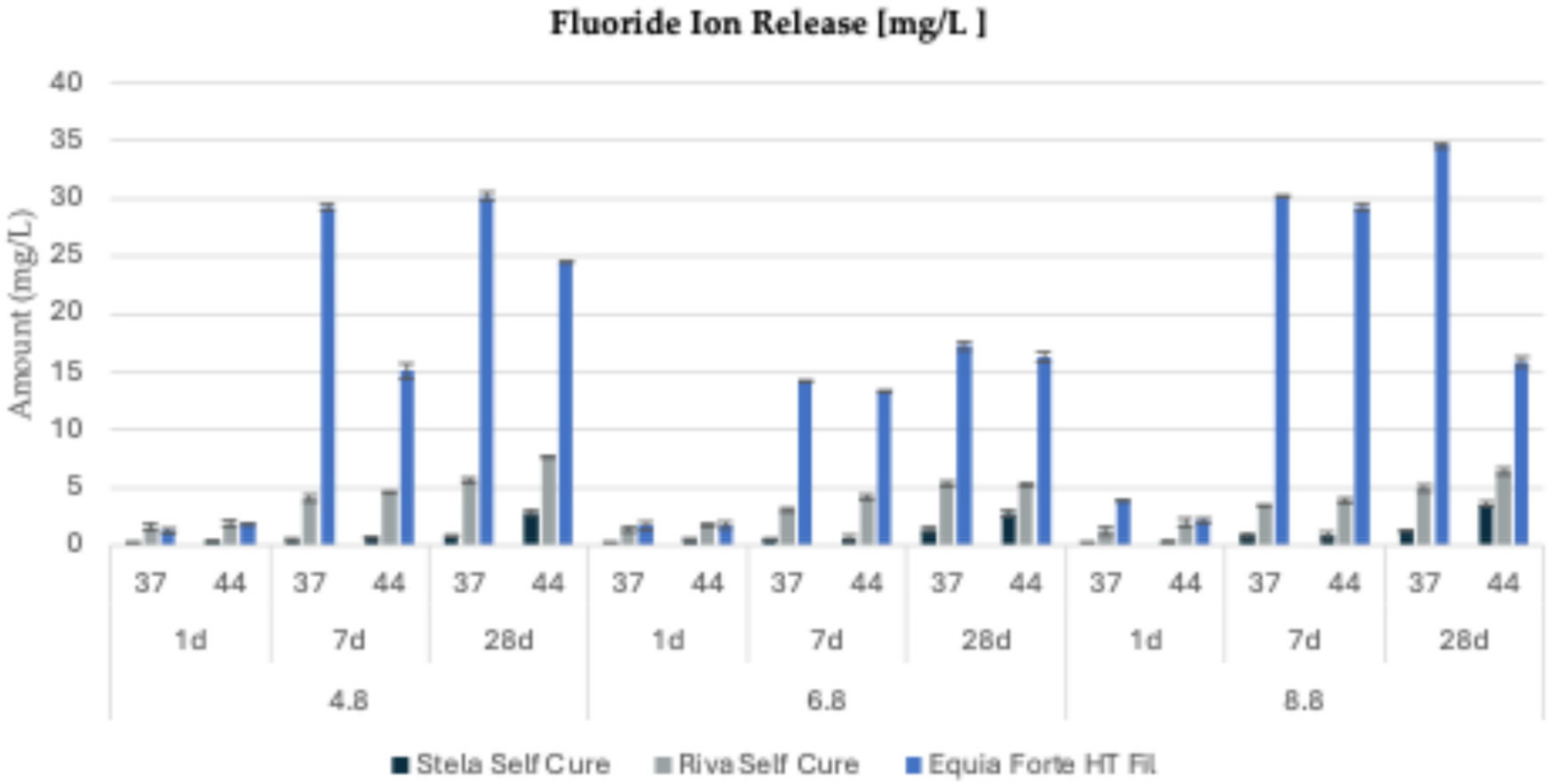
Fluoride ion release (mg/L) from three tested restorative materials (*Stela Self Cure*, *Riva Self Cure*, and *Equia Forte HT Fil*) over different time points (1 day, 7 days, and 28 days) at three different pH levels (4.8, 6.8, and 8.8) and two temperatures (44 and 37°C).

**Figure 4:**
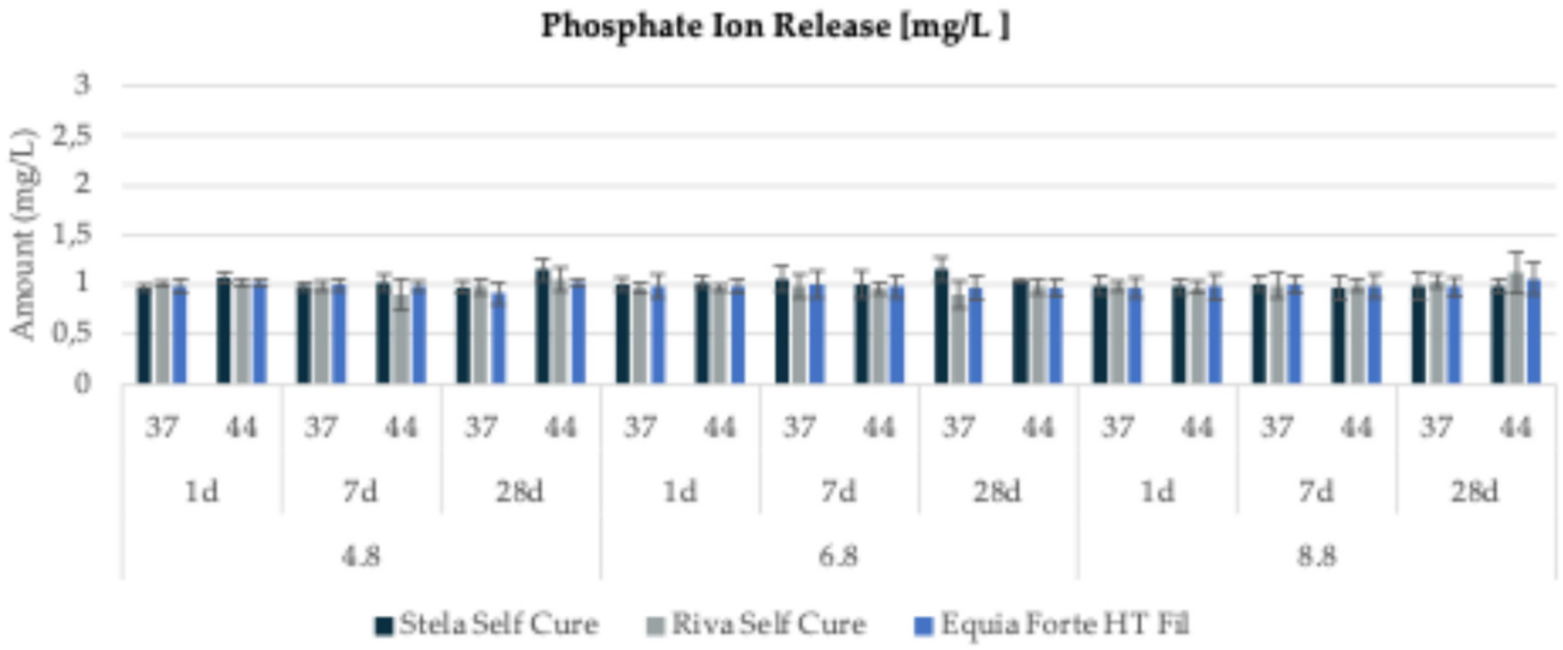
Phosphate ion release (mg/L) from three tested restorative materials (*Stela Self Cure*, *Riva Self Cure*, and *Equia Forte HT Fil*) over different time points (1 day, 7 days, and 28 days) at three different pH levels (4.8, 6.8, and 8.8) and two temperatures (44 and 37°C).

**Table 3:** Mean fluoride ion concentration along with standard deviations (SD) recorded for each tested material under three distinct pH conditions (4.8, 6.8 and 8.8), measured at two temperatures (37°C and 44°C) and across three observation periods (1 day, 7 days, and 28 days).

Buffer solution pH	Time	T (°C)	Fluoride Ion Release [mg/L]		
			Stela Self Cure	Riva Self Cure	Equia Forte HT Fil
4.8	1d	37	0.28 ± 0.07	1.61 ± 0.31	1.27 ± 0.22
		44	0.34 ± 0.06	1.94 ± 0.27	1.90 ± 0.08
	7d	37	0.41 ± 0.12	4.10 ± 0.33	29.27 ± 0.27
		44	0.70 ± 0.07	4.57 ± 0.09	15.08 ± 0.60
	28d	37	0.78 ± 0.07	5.62 ± 0.20	30.15 ± 0.33
		44	2.82 ± 0.25	7.67 ± 0.09	24.48 ± 0.04
6.8	1d	37	0.22 ± 0.04	1.41 ± 0.24	1.70 ± 0.34
		44	0.39 ± 0.15	1.72 ± 0.15	1.77 ± 0.33
	7d	37	0.64 ± 0.03	3.06 ± 0.16	14.28 ± 0.05
		44	0.57 ± 0.26	4.17 ± 0.22	13.33 ± 0.13
	28d	37	1.39 ± 0.23	5.33 ± 0.17	17.30 ± 0.36
		44	2.80 ± 0.26	5.28 ± 0.16	16.27 ± 0.40
8.8	1d	37	0.23 ± 0.06	1.29 ± 0.33	3.89 ± 0.09
		44	0.29 ± 0.10	1.98 ± 0.32	2.18 ± 0.23
	7d	37	0.83 ± 0.16	3.42 ± 0.07	30.15 ± 0.10
		44	0.84 ± 0.30	3.92 ± 0.23	29.23 ± 0.33
	28d	37	1.34 ± 0.05	4.93 ± 0.28	34.59 ± 0.23
		44	3.55 ± 0.24	6.41 ± 0.34	15.89 ± 0.40

**Table 4:** Mean phosphate ion concentration along with standard deviations (SD) recorded for each tested material under three distinct pH conditions (4.8, 6.8 and 8.8), measured at two temperatures (37°C and 44°C) and across three observation periods (1 day, 7 days, and 28 days).

Buffer solution pH	Time	T (°C)	PO_4_^3−^ Release [mg/L]		
			Stela Self Cure	Riva Self Cure	Equia Forte HT Fil
4.8	1d	37	0.97 ± 0.03	1.01 ± 0.02	0.98 ± 0.07
		44	1.07 ± 0.05	1.02 ± 0.03	1.02 ± 0.04
	7d	37	0.98 ± 0.04	0.99 ± 0.05	1.00 ± 0.06
		44	1.02 ± 0.08	0.90 ± 0.16	0.99 ± 0.05
	28d	37	0.97 ± 0.06	0.98 ± 0.08	0.91 ± 0.11
		44	1.15 ± 0.11	1.06 ± 0.12	1.02 ± 0.03
6.8	1d	37	1.00 ± 0.07	0.97 ± 0.05	0.98 ± 0.12
		44	1.02 ± 0.06	0.97 ± 0.04	0.98 ± 0.07
	7d	37	1.06 ± 0.13	0.98 ± 0.12	1.00 ± 0.14
		44	1.00 ± 0.14	0.96 ± 0.06	0.98 ± 0.11
	28d	37	1.16 ± 0.12	0.90 ± 0.14	0.97 ± 0.12
		44	1.04 ± 0.02	0.98 ± 0.08	0.97 ± 0.08
8.8	1d	37	0.99 ± 0.09	0.98 ± 0.05	0.97 ± 0.10
		44	0.98 ± 0.08	0.97 ± 0.06	0.98 ± 0.13
	7d	37	1.01 ± 0.08	0.99 ± 0.13	1.00 ± 0.08
		44	0.96 ± 0.12	0.99 ± 0.06	0.99 ± 0.12
	28d	37	0.98 ± 0.14	1.04 ± 0.07	0.98 ± 0.09
		44	0.99 ± 0.07	1.12 ± 0.20	1.06 ± 0.16

For *Equia Forte HT Fil*, the highest fluoride release (34.59 ± 0.63 mg/L) was recorded after 28 days in a basic environment (pH = 8.8) at 37°C (Figure 3). Specifically, cumulative fluoride release ranged from 1.27 ± 0.22 to 30.15 ± 0.43 mg/L at pH 4.8 (mean value: 17.02 ± 13.10 mg/L), from 1.70 ± 0.34 to 17.30 ± 0.36 mg/L at pH 6.8 (mean: 10.78 ± 7.14 mg/L), and from 2.18 ± 0.23 to 34.59 ± 0.63 mg/L at pH 8.8 (mean: 19.32 ± 14.09 mg/L). Regarding phosphate ion release (Figure 4), values ranged from 0.91 ± 0.11 mg/L (at pH 4.8, 37°C, after 28 days) to 1.06 ± 0.16 mg/L (at pH 8.8, 44°C, after 28 days).

For *Stela Self Cure*, the highest fluoride release (3.55 ± 0.24 mg/L) occurred at 44°C after 28 days in a basic environment (pH = 8.8) (Figure 3). Fluoride concentrations varied from 0.28 ± 0.07 to 2.82 ± 0.25 mg/L at pH 4.8 (mean: 0.89 ± 0.97 mg/L), from 0.22 ± 0.04 to 2.80 ± 0.26 mg/L at pH 6.8 (mean: 1.00 ± 0.96 mg/L), and from 0.23 ± 0.06 to 3.55 ± 0.24 mg/L at pH 8.8 (mean: 1.18 ± 1.23 mg/L). Phosphate ion release followed a relatively stable trend, ranging from 0.96 ± 0.12 mg/L (at pH 8.8, 44°C, after 7 days) to 1.16 ± 0.13 mg/L (at pH 6.8, 37°C, after 28 days) (Figure 4).

For *Riva Self Cure*, the highest fluoride release (7.67 ± 0.49 mg/L) was observed after 28 days at 44°C in an acidic environment (pH = 4.8) (Figure 3). Fluoride release ranged from 1.61 ± 0.31 to 7.67 ± 0.49 mg/L at pH 4.8 (mean: 4.25 ± 2.28 mg/L), from 1.41 ± 0.34 to 5.33 ± 0.47 mg/L at pH 6.8 (mean: 3.50 ± 1.71 mg/L), and from 1.29 ± 0.33 to 6.41 ± 0.44 mg/L at pH 8.8 (mean: 3.66 ± 1.88 mg/L). In terms of phosphate ion release (Figure 4), values ranged from 0.90 ± 0.16 mg/L (at pH 6.8, 44°C, after 7 days) to 1.12 ± 0.20 mg/L (at pH 8.8, 44°C, after 28 days) (Figure 4).

Overall, fluoride and phosphate ion release patterns varied depending on pH, temperature, and observation time. While some materials, such as Stela Self Cure, demonstrated increased fluoride release at higher temperatures and pH levels, others, like Equia Forte HT Fil and Riva Self Cure, exhibited more pronounced fluoride release in acidic or basic environments. In contrast, phosphate ion release remained relatively consistent across all materials. These findings highlight the distinct release behaviors of the tested materials, emphasizing their potential variability in clinical applications based on environmental conditions.

### 3.3 Calcium Ion release

**Figure 5:**
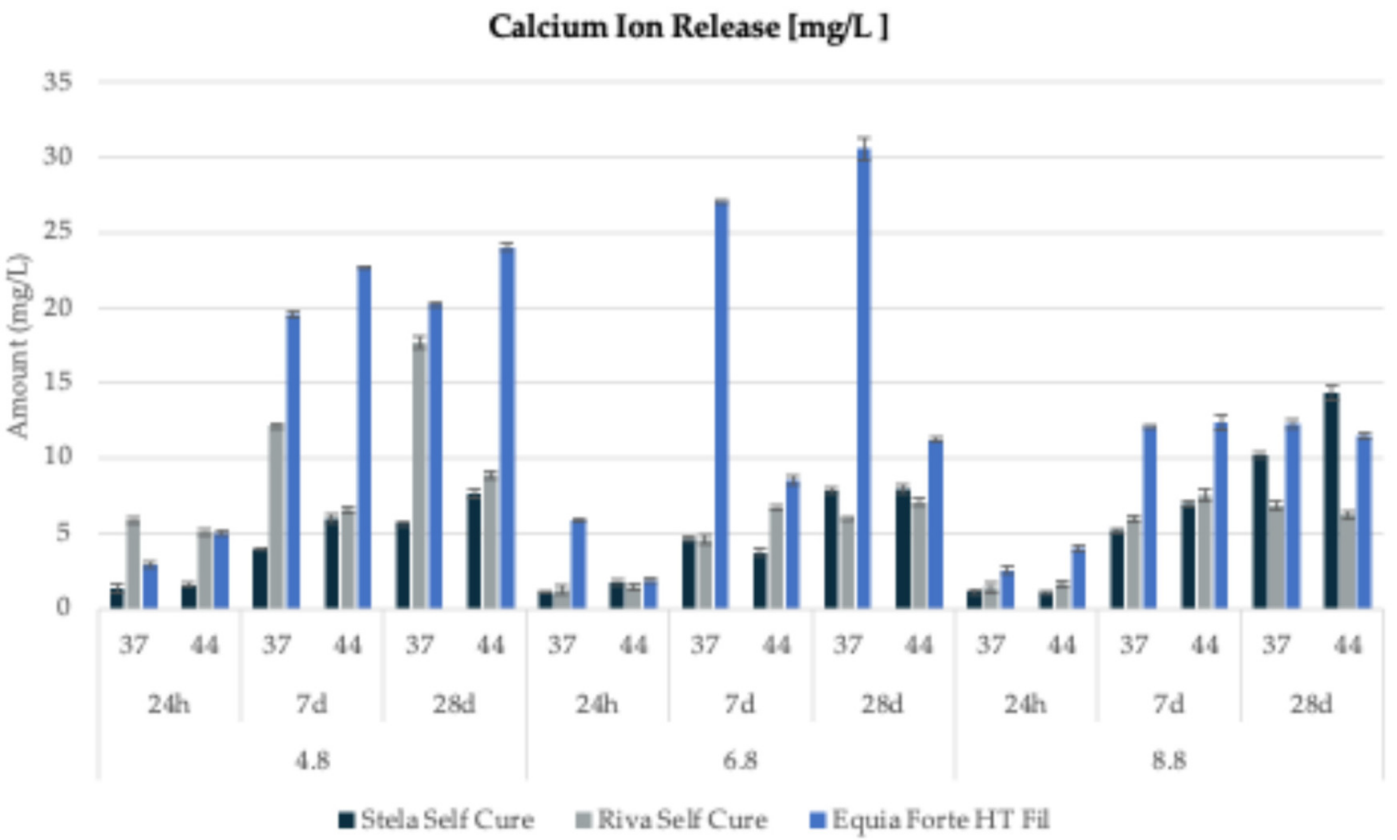
Calcium ion release (mg/L) from three tested restorative materials (*Stela Self Cure*, *Riva Self Cure*, and *Equia Forte HT Fil*) over different time points (1 day, 7 days, and 28 days) at three different pH levels (4.8, 6.8, and 8.8) and two temperatures (44 and 37°C).

The calcium ion (Ca^2+^) release for all tested materials under three different pH conditions, two temperatures (37°C and 44°C), and three observation periods (1 day, 7 days, and 28 days) is presented in Figure 5 and detailed in Table 5.

**Table 5:** Mean calcium ion concentration along with standard deviations (SD) recorded for each tested material under three distinct pH conditions (4.8, 6.8 and 8.8), measured at two temperatures (37°C and 44°C) and across three observation periods (1 day, 7 days, and 28 days).

Buffer solution pH	Time	T (°C)	Ca^2+^ Release [mg/L]		
			Stela Self Cure	Riva Self Cure	Equia Forte HT Fil
4.8	24h	37	1.30 ± 0.28	5.87 ± 0.16	2.89 ± 0.22
		44	1.57 ± 0.18	5.12 ± 0.21	5.02 ± 0.13
	7d	37	3.95 ± 0.09	12.15 ± 0.21	19.55 ± 0.22
		44	5.97 ± 0.27	6.55 ± 0.20	22.68 ± 0.08
	28d	37	5.64 ± 0.18	17.69 ± 0.42	20.24 ± 0.17
		44	7.68 ± 0.29	8.88 ± 0.28	24.05 ± 0.26
6.8	24h	37	1.05 ± 0.05	1.25 ± 0.32	5.89 ± 0.09
		44	1.69 ± 0.20	1.44 ± 0.17	1.85 ± 0.16
	7d	37	4.60 ± 0.14	4.58 ± 0.33	27.06 ± 0.14
		44	3.73 ± 0.24	6.67 ± 0.14	8.54 ± 0.30
	28d	37	7.86 ± 0.20	5.94 ± 0.09	30.6 ± 0.73
		44	7.94 ± 0.25	7.08 ± 0.23	11.23 ± 0.16
8.8	24h	37	1.12 ± 0.13	1.40 ± 0.38	2.56 ± 0.25
		44	1.04 ± 0.11	1.67 ± 0.21	4.02 ± 0.22
	7d	37	5.16 ± 0.15	5.95 ± 0.19	12.09 ± 0.09
		44	6.98 ± 0.19	7.54 ± 0.43	12.39 ± 0.47
	28d	37	10.23 ± 0.17	6.88 ± 0.30	12.27 ± 0.30
		44	14.35 ± 0.45	6.22 ± 0.25	11.5 ± 0.22

For *Stela Self Cure*, the highest calcium release (14.35 ± 0.45 mg/L) was detected after 28 days at 44°C in a basic environment (pH = 8.8). The recorded concentrations varied from 1.31 ± 0.28 to 7.68 ± 0.29 mg/L at pH 4.8 (mean: 4.35 ± 2.55 mg/L), from 1.05 ± 0.05 to 7.94 ± 0.25 mg/L at pH 6.8 (mean: 4.48 ± 2.95 mg/L), and from 1.04 ± 0.11 to 14.35 ± 0.45 mg/L at pH 8.8 (mean: 6.48 ± 5.22 mg/L) (Figure 5).

For *Riva Self Cure*, calcium release ranged from 5.12 ± 0.21 to 17.69 ± 0.42 mg/L at pH 4.8 (mean: 9.38 ± 4.81 mg/L), from 1.25 ± 0.32 to 7.08 ± 0.23 mg/L at pH 6.8 (mean: 4.49 ± 2.58 mg/L), and from 1.40 ± 0.38 to 7.54 ± 0.43 mg/L at pH 8.8 (mean: 4.94 ± 2.70 mg/L). The highest calcium release was recorded in an acidic environment at 37°C after 28 days (Figure 5).

Finally, for *Equia Forte HT Fil*, the highest calcium concentrations were observed in a neutral medium (pH 6.8), with a maximum release of 30.60 ± 0.73 mg/L after 28 days at 37°C (Figure 5). The release ranged from 2.89 ± 0.22 to 24.05 ± 0.26 mg/L in acidic conditions (mean: 15.74 ± 9.29 mg/L), from 1.85 ± 0.16 to 30.60 ± 0.73 mg/L in neutral conditions (mean: 14.20 ± 11.81 mg/L), and from 2.56 ± 0.25 to 12.39 ± 0.47 mg/L in basic conditions (mean: 9.14 ± 4.56 mg/L).

In summary, the evaluation of calcium ion release across the tested dental materials revealed notable variations in their release patterns. *Stela Self Cure* exhibited the highest calcium release under basic conditions, whereas *Equia Forte HT Fil* consistently showed lower calcium concentrations across all tested environments, indicating greater stability. These findings underscore the diverse release behaviors of dental materials, emphasizing the need for careful material selection based on specific clinical applications and their long-term effects on the oral environment.

### 3.4 Results of statistical analysis

The statistical analysis demonstrated that ion release was significantly influenced by key factors, including the acidity of the medium, temperature, and exposure duration, with distinct trends among the tested materials. Specifically, fluoride release was notably affected by pH (*p* = 0.009) and exposure time (*p* < 0.001), with higher concentrations observed under extreme pH conditions (acidic or basic) and over extended periods. Calcium release exhibited a negative correlation with pH (*p* < 0.001), indicating increased solubilization in acidic environments, while exposure time had a significant positive effect (*p* < 0.001), reflecting a continuous release pattern. In contrast, phosphate release was predominantly influenced by exposure time (*p* < 0.001), whereas pH and temperature had no statistically significant impact (*p* > 0.05), suggesting a more stable release profile compared to other ions. Additionally, multivariate analysis confirmed the substantial role of both pH and exposure duration in ion release dynamics, with significant variations observed among the tested materials (*p* < 0.05). These results emphasize the importance of environmental conditions in the selection of restorative materials, as their performance may be affected by fluctuations in pH and temperature within the oral cavity.

## 4 Discussion

The global demographic shift toward an increasingly aging population has significant implications for oral health (41). Older adults often present with complex clinical needs, including a high prevalence of periodontal disease, root caries, and non-carious cervical lesions (NCCLs), particularly those classified as Class V (42). Epidemiological data show that periodontitis is highly prevalent among the elderly and often leads to gingival recession and root surface exposure (43). In patients with periodontal attachment loss, Class V lesions are frequently associated with root dentin exposure, a substrate that is more prone to demineralization due to its lower mineral content and increased permeability compared to enamel (44). Therefore, restorative approaches in such cases must be minimally invasive, highly adhesive to root dentin, and ideally, capable of releasing ions that can promote remineralization and reduce the risk of recurrent lesions (45). The management of these lesions in geriatric patients requires restorative strategies that are not only mechanically stable but also biologically active, providing long-term therapeutic benefits.

In this context, restorative materials such as conventional glass ionomer cements (GICs), resin-modified glass ionomer cements (RMGICs), and composite resins are frequently employed in clinical practice (23). Traditional GICs offer chemical adhesion to dental tissues, biocompatibility, and sustained fluoride release, which is critical in preventing secondary caries. However, their mechanical properties, including wear resistance and fracture toughness, are often inferior to those of composite resins (46). RMGICs combine the benefits of fluoride release with improved physical characteristics and reduced sensitivity to moisture during setting. Composite resins exhibit superior esthetics and mechanical strength but lack inherent ion release capabilities unless modified with bioactive components (47).

A critical drawback of composite resins, especially in Class V lesions, is the polymerization shrinkage stress, which can compromise marginal integrity, particularly in lesions located on sclerotic or thin dentin (48). This stress can lead to gap formation, microleakage, and ultimately, restoration failure. In elderly patients with reduced pulp vitality and periodontally compromised teeth, it is particularly important to minimize the risk of mechanical stress and microinfiltration (49–51).

This *in vitro* study evaluated the ionic release profiles of three restorative materials: *Equia Forte HT Fil*, a high-viscosity glass hybrid restorative system, *Riva Self Cure*, a conventional GIC, and *Stela Self Cure*, a self-curing based material with bioactive potential. The findings indicate substantial differences in the ionic release behavior among these materials under both neutral (pH 6.8) and acidic (pH 4.0) conditions, which simulate physiological and cariogenic oral environments, respectively. So, the null hypothesis of this study was rejected. Specifically, *Equia Forte HT Fil* showed the highest fluoride and calcium ion release, especially in acidic (pH 4.8) and neutral (pH 6.8) environments. This behavior can be attributed to its formulation, which includes a high percentage of fluoride-containing aluminosilicate glass particles and polyacrylic acid, designed to respond to pH changes through ion exchange mechanisms. The presence of silicate-reinforced glass fillers likely enhances its solubility and ion mobility under low pH, explaining the increased release under demineralizing conditions. Additionally, temperature elevation to 44°C likely facilitated ion diffusion by increasing molecular activity within the glass-ionomer matrix.

*Riva Self Cure* also exhibited fluoride release, though at lower levels compared to *Equia Forte HT Fil*, and its calcium and phosphate release was minimal. Its reduced calcium and phosphate output may stem from differences in its glass composition and the absence of additional reinforcing phases. However, it maintained a stable fluoride release, particularly in acidic and warm conditions, due to the presence of tartaric acid and fluoride-rich glass. The lower overall solubility of *Riva Light Cure* matrix may account for its slower ion diffusion, but it still offers meaningful anticariogenic potential in a clinical setting. Furthermore, its low polymerization shrinkage makes it ideal in treating Class V lesions in areas of gingival recession and root exposure, particularly in periodontally involved teeth.

*Stela Self Cure*, a resin-based material containing UDMA, GDMA, and ytterbium fluoride, exhibited limited ion release across all conditions. Its hydrophobic matrix restricts water penetration and ion diffusion, resulting in negligible calcium and phosphate release. Although a modest amount of fluoride was released, likely from embedded ytterbium fluoride particles, this was not significantly influenced by pH or temperature. While it may still offer some degree of caries prevention through fluoride, its clinical impact on promoting remineralization is potentially less significant than that of materials like *Equia Forte HT Fil*. However, its superior mechanical performance and handling properties may compensate for this limitation in restorations requiring higher strength and durability.

The influence of pH was particularly pronounced across all materials, with acidic conditions generally increasing ion solubility and release. This is especially relevant in elderly patients with xerostomia or acidic oral environments. Similarly, the elevated temperature (44°C) accelerated ion mobility, mimicking thermal fluctuations from dietary habits (e.g., hot beverages). Lastly, the immersion period played a consistent role, with longer exposure times resulting in cumulative ion release, particularly for fluoride and calcium.

The interplay between pH and ionic release is particularly relevant in the context of elderly patients, who often exhibit fluctuating oral pH due to salivary alterations, polypharmacy, and dietary factors (52). Materials that respond to acidic challenges by increasing ion release, such as *Equia Forte HT Fil*, can provide a protective buffer during episodes of demineralization. This dynamic response represents a valuable clinical feature in managing high-risk geriatric patients (38, 53).

These findings emphasize the importance of considering both material composition and environmental factors when selecting restorative solutions for elderly patients. Materials like *Equia Forte HT Fil*, which combine ion-releasing properties with responsiveness to acidic and thermal challenges, are particularly suitable for managing high-risk cases, including Class V lesions and root caries.

It is also important to acknowledge that other contemporary restorative materials, such as giomers, alkasites, and resin-based systems containing surface pre-reacted glass-ionomer (S-PRG) fillers, have also been developed with bioactive properties. Giomers combine the esthetics and handling of composite resins with fluoride release from S-PRG technology, although their ion release is typically lower than that of conventional GICs (54). Alkasites are resin-based materials with alkaline glass fillers that release fluoride, calcium, and hydroxide ions, contributing to remineralization and pH buffering (55). S-PRG fillers, present in both giomers and other materials, allow multi-ion release and offer anti-demineralization and mild antibacterial effects (56). While these materials were not included in the present study, their increasing use in clinical practice underscores the need for future comparative studies evaluating their ion release behavior and bioactive potential alongside GIC-based systems.

Despite the promising outcomes, this study presents some limitations. First, the *in vitro* nature of the experiment does not fully replicate the complexity of the oral environment, where biofilm activity, salivary flow, enzymatic degradation, and mechanical stress from mastication may alter material performance. Additionally, the evaluation focused solely on ion release, without assessing other important factors such as polymerization kinetics, mechanical durability over time, or patient-related outcomes. The observation period was limited, and long-term ion release kinetics remain to be fully elucidated. Furthermore, another important limitation is the relatively small sample size (*n* = 3 per group), which, although determined to be statistically adequate through power analysis, may limit the generalizability of the findings. Future studies with larger sample sizes are recommended to confirm and expand upon these results under more clinically representative conditions. In addition, upcoming trials should incorporate *in vivo* studies, extended observation periods, and comparative clinical trials to validate the therapeutic relevance of ion-releasing materials in elderly populations. Investigating the behavior of these materials in the presence of periodontal inflammation and in root surfaces affected by dentinal sclerosis would also provide valuable clinical insight.

## 5 Conclusions

This *in vitro* study offers preliminary evidence on the ion release profiles of three restorative materials, *Equia Forte HT Fil*, *Riva Self Cure*, and *Stela Self Cure* when exposed to varying pH and temperature conditions. Among them, *Equia Forte HT Fil* showed higher cumulative release of calcium and fluoride ions, which may contribute to its potential remineralizing effect. All tested materials exhibited some degree of bioactivity, with ion release behavior influenced by environmental conditions such as acidity, temperature, and immersion time. Given the increased prevalence of root caries and non-carious cervical lesions in the elderly, restorative materials capable of releasing bioactive ions may offer therapeutic benefits in this vulnerable population. However, considering the exploratory nature of this study and its *in vitro* limitations, further *in vivo* and clinical investigations are essential to validate the clinical relevance of these findings and to better understand the role of ion-releasing restorative materials in managing high-caries-risk elderly patients.

## Data Availability

The original contributions presented in the study are included in the article/Supplementary Material, further inquiries can be directed to the corresponding author.
